# MaxReport: An Enhanced Proteomic Result Reporting Tool for MaxQuant

**DOI:** 10.1371/journal.pone.0152067

**Published:** 2016-03-22

**Authors:** Tao Zhou, Chuyu Li, Wene Zhao, Xinru Wang, Fuqiang Wang, Jiahao Sha

**Affiliations:** 1 State Key Laboratory of Reproductive Medicine, Nanjing Medical University, Nanjing, 210029, PR China; 2 Analytical and Testing Center, Nanjing Medical University, Nanjing, 210029, PR China; Moffitt Cancer Center, UNITED STATES

## Abstract

MaxQuant is a proteomic software widely used for large-scale tandem mass spectrometry data. We have designed and developed an enhanced result reporting tool for MaxQuant, named as MaxReport. This tool can optimize the results of MaxQuant and provide additional functions for result interpretation. MaxReport can generate report tables for protein N-terminal modifications. It also supports isobaric labelling based relative quantification at the protein, peptide or site level. To obtain an overview of the results, MaxReport performs general descriptive statistical analyses for both identification and quantification results. The output results of MaxReport are well organized and therefore helpful for proteomic users to better understand and share their data. The script of MaxReport, which is freely available at http://websdoor.net/bioinfo/maxreport/, is developed using Python code and is compatible across multiple systems including Windows and Linux.

## Introduction

MaxQuant is a free academic software developed for interpreting large-scale proteomic data based on tandem mass spectrometry (MS/MS) [1]. It was originally a quantifying package for isotope labelling and required the identification results generated by the commercial search engine Mascot [2]. In 2011, MaxQuant integrated its own peptide search engine, Andromeda, which is based on a probabilistic scoring model [3]. MaxQuant is highly flexible and has been widely applied in various proteomic studies including peptide identification, modification assignment, isotope labelling quantification and label-free quantification [4–7]. However, the outputs of MaxQuant comprise of numerous tab separated text files (table files) with complex columns. It is hard for biomedical researchers who are not familiar with bioinformatics to process and share the results. For protein N-terminal modifications such as N-terminal acetylation sites, MaxQuant only incorporates them for peptide searches without generating reporting tables like other post-translational modifications (PTMs), such as phosphorylation sites. In addition, MaxQuant currently does not support isobaric labelling quantification, although its latest version (1.5.x) can identify labelled peptides and extract raw intensities of reporter ions. On the other hand, many third-party tools can provide enhanced or additional functions for the original proteomic software. For example, Mascot Percolator was developed to interface Mascot results with Percolator, which improves peptide identification based on machine learning algorithm [8]. IQuant is a post-processing software for isobaric labelling quantification, which also needs the identification results generated by Mascot [9].

Considering these factors, we aimed to design and develop an enhanced proteomic result reporting tool for MaxQuant, called as MaxReport. The main objectives of MaxReport are to optimize the results of MaxQuant and to provide additional functions for protein N-terminal modifications, isobaric labelling quantification, and descriptive statistical analyses.

## Materials and Methods

### MaxReport workflow

**Fig 1:**
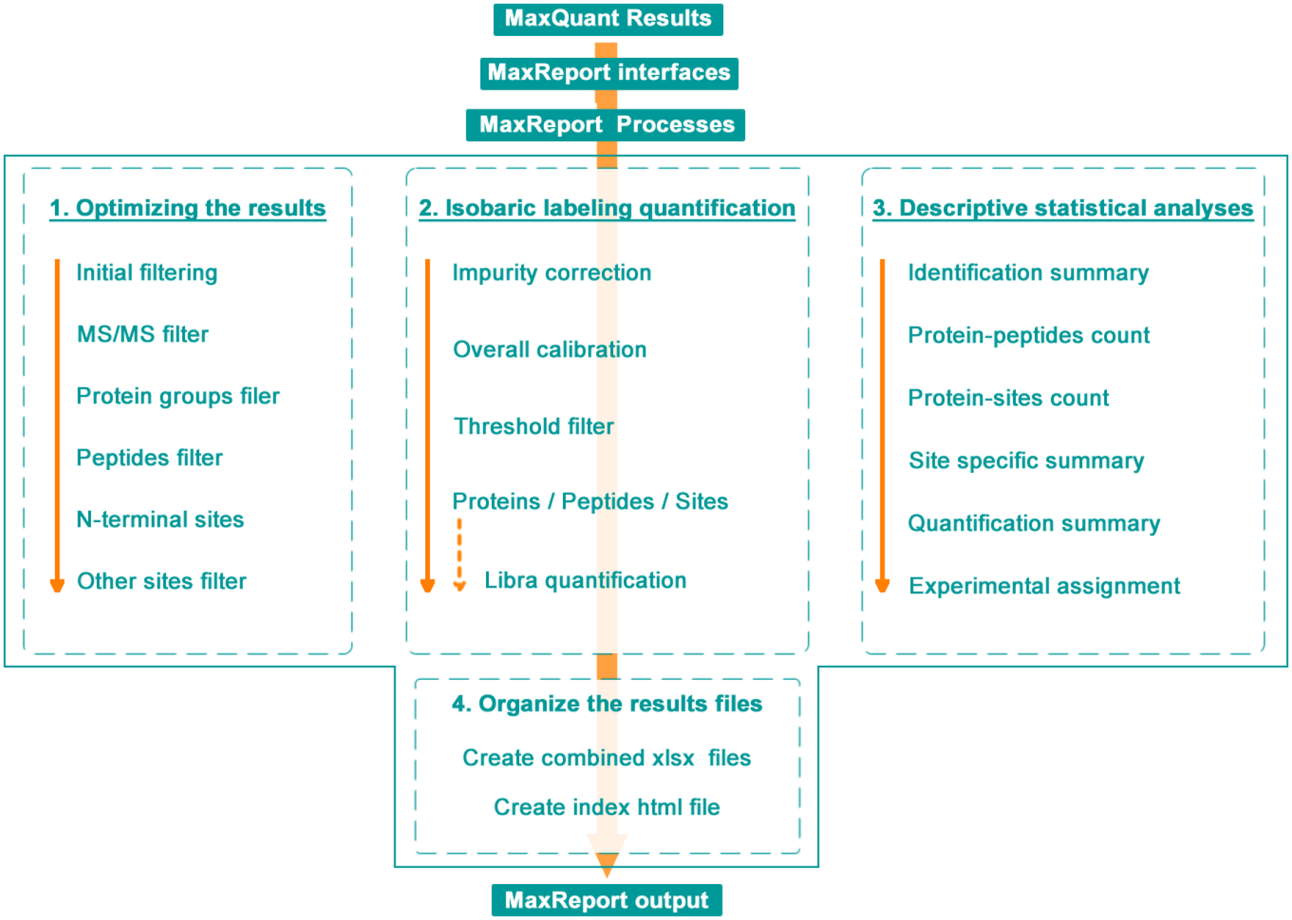
Overview of the MaxReport workflow.

MaxReport takes the MaxQuant project directory as input. The workflow of the following processes is shown in Fig 1, which comprises of four parts as detailed below:

MaxReport processes the input MaxQuant results in four parts to provide optimized results and additional functions.

#### (1). Optimizing the results

There are many table files in the "combined/txt" folder of a MaxQuant output directory. Each table file consists of plenty of columns. Thus, our first goal was to select and compress the result files. Considering the minimum reporting guidelines of the Human Proteome Organization Proteomics Standards Initiative (HUPO-PSI) [10], we selected and reported the results at four levels: protein, peptide, modified site and MS/MS scans, which correspond to "proteinGroups.txt" file, "peptides.txt" file, possible "XXXSites.txt" (XXX stands for a PTM name) files, and "msms.txt" file respectively. Since the column titles and scoring methods for generating the result files are slightly various in different versions of MaxQuant, we designed a reporting configuration file for extracting the minimum columns according to specified titles. Two types of titles are thereby defined: exact titles (exact matching mode) and keyword titles (keyword matching mode). Keyword titles can be used to extract experimental specific columns, PTM sites and other varying titles. The name of protein N-terminal modifications (or other types of PTMs that MaxQuant does not report) should be specified in the configuration file for further generation of site tables. Once the reporting configuration file is loaded, MaxReport starts from the protein group table for initial filtering. First, reverse and contaminant hits are removed. Many protein accessions may be grouped as a protein group due to shared peptides. We only keep those protein accessions mapped with the largest number of unique peptides and total peptides in a group for minimum reporting. Then, the first protein accession gets annotated (extract gene name and protein description information) using the fasta sequence file and the corresponding rule parsing file. MaxReport 2.0 starts to support multiple sequence files. The rule files and the corresponding fasta files should be provided in the same order for MaxReport to load all sequences correctly. If a peptide or site is mapped to multiple protein groups, the first protein of the protein group with the best score also gets annotated. Protein N-terminal sites are recognized based on the "msms.txt" table file, and then the peptide and protein group information are extracted according to MS/MS identities in order to construct a site table. For PTMs reporting, we extracted the sequence window of a defined length (six residues to both terminals by default) for each site, including N-terminal modifications, using the best scored protein sequence. In addition, many publishing journals require spectra image files for publication as a supplementary data. However, a large-scale proteomic study usually generates thousands of MS/MS scans, which creates extremely large image files for these scans. Thus, it is preferable to provide the annotated spectra including peptide match, fragmentation annotations, ion intensities, and matching scores as a text table file that can be selected from "msms.txt" with a smaller file size.

#### (2). Isobaric labelling quantification

Isobaric reagents such as Isobaric tags for relative and absolute quantitation (iTRAQ) [11] and tandem mass tag (TMT) [12] are widely used for protein quantification. Several tools such as IQuan [9] and IsobariQ [13] have been developed for isobaric labelling quantification. These tools are mainly post-processing software for the commercial search engine Mascot. One major limitation of these tools is the need to specify a reference reporter for generating expression ratios. For example, for TMT6 labelling, we can only get relative expression ratios such as TMT-127/TMT-126 or TMT-128/TMT-126 for each peptide or protein. However, if the peptide or protein expression in one sample is zero (this is common for a gene knockout versus wild-type experimental design), ratios cannot be calculated or the tool crashes completely. Thus, we employed a revised Libra algorithm, a module within the trans-proteomic pipeline (TPP) [14], to perform quantification for MaxReport in order to provide relative expression values directly for each reporter at the protein, peptide or site level. Following the guidelines for reporting quantitative proteomic results [15], MaxReport first extracted experimental design information (replication assignment) by using the "summary.txt" file in the "combined/txt" folder or by a specified experimental design template file. Each replication was quantified separately and then merged according to the mapping identities. The raw intensities of each reporter ions were obtained from "msms.txt" file. Due to the existence of isotopic overlap [16], the reporter intensities are recommended to be corrected by using a correction matrix file. Then, the overall intensities of each reporter were calibrated to be equal. Once the low threshold values were removed, unique peptides or all peptides were used for further quantification purposes. Since MaxQuant also reports ambiguous sites (the corresponding peptide was calculated to be modified; however the localization of the modified site was not determined due to poor fragmentation ions), we therefore classified sites into specific (with a localization probability larger than 0.5 by default) and ambiguous sites. Only specific sites were used for site quantification. Spectra were grouped and assigned to each protein, peptide or site for separate Libra quantification. We have successfully combined MaxQuant results and Libra algorithm to identify differentially expressed proteins in a previous study [17]. Briefly, the ion intensity of each reporter ion is normalized by the sum of intensities for every spectrum. After removing the outliers (which differ from the mean value by more than 1.96 sigma) in a group of spectra, the median as well as mean intensity values of each reporter ion are recalculated and reported as the final relative expression values. We also provided standard deviation for each reporter ion.

#### (3). Descriptive statistical analyses

To provide an overview of the reporting results, MaxReport further performs general descriptive statistical analyses and creates histogram and pie figures for visualization. First, the overall identified protein groups, peptides and spectra are counted. For each type of PTM, the counts of the sites and the corresponding spectra, peptides and protein groups are summarized. For protein groups, the distribution of all peptides and unique peptides numbers is calculated. For each PTM, the distribution of site numbers is also calculated. On detecting reporter ions for isobaric labelling, the overall calibration factors for each channel are exported. The total numbers of the quantified protein groups, peptides and PTM sites are also provided. If the experimental replications are detected, MaxReport will generate replication assignment files for protein group, peptide, PTM sites and MS/MS scans.

#### (4). Organize the results files

MaxReport creates a folder called "maxreport" in the input directory to store all of the result files. An index page ("index.html") is created to provide well organized guides for the users to conveniently find and understand the results. The index page is divided into six parts: (i). "Input parameters" shows the input command line. (ii). "Identification results" lists the location of result files. (iii). "Summary and descriptive statistics" shows the location of statistical analyses files and the corresponding figures. (iv). "Quantification based on isobaric labelling" shows the location of quantification result files and the corresponding statistical figures. (v). "Miscellaneous" provides extracted protein sequences and MS/MS scan identities based on the optimized results. (vi). "Log file" indicates the location of the log file that records the time and notes for all key processes. Error messages are also displayed in the file when the tool crashes. The index page is automatically opened once all the processes are completed. If there is no index file generated, the users should check the log file for errors. For complex questions, the users can report the errors via our online feedback system for technical supports.

### Example data preparation

Two published datasets were used to test the application of MaxReport. One dataset was derived from a large-scale proteome study of macaque sperm with four technical replicates [4]. The raw data was searched by MaxQuant version 1.3.0.5 using the Ensembl protein sequences (36,384 sequences) of Macaca mulatta (MMUL_1.0) [18]. For protein identification, enzyme specificity was set to be fully cleaved by trypsin. The maximum number of missed cleavage sites permitted was two, and the minimum peptide length required was six. Carbamidomethyl (C) was set as a fixed modification. Variable modifications were oxidation (M) and N-terminal Acetylation (Protein N-term). The false discovery rate (FDR) of identification was estimated by searching database with reversed sequences. The peptide and protein FDRs were all set to 0.01.

The other dataset was a TMT6 labelling standard experiment spiked with four exogenous proteins of known expression ratios [19]. The raw data (PXD000001) was downloaded from the Proteomics Identifications (PRIDE) database via ProteomeXchange [20] and reprocessed with MaxQuant (version 1.5.2.8). Protein sequences of Erwinia carotovora (3,827 entries) and standard proteins (4 entries) for database search were downloaded from UniProt database (release 2015_06) [21]. The identification parameters are the same with the above dataset. In addition, TMT6 plex labeling was selected for extracting intensities of reporter ions.

## Results and Discussions

### Software availability

The current version of MaxReport tool was developed and tested by using ActivePython (version 2.7.5.6) under a Windows system. In addition, NumPy (version 1.8.1) was used for mathematical calculation. Pygal (version 1.7.0) was used for creating statistical figures in scalable vector graphics (SVG) format. To generate combined result tables in Excel format (xlsx), XlsxWriter (version 0.6.4) was used. wxPython (version 3.0) was applied for GUI development. The latest MaxReport package (version 2.1) and associated resources can be downloaded for free at http://websdoor.net/bioinfo/maxreport/. MaxReport 2.1 package contains MaxReport_CMD (core program with command line interface), MaxReport_GUI (graphical user interface for MaxReport_CMD), a detailed documentation in portable document format (PDF), and resource files for configurations. As Python is a cross-platform programming language used widely for developing scientific tools [22], the MaxReport scripts (source code) could be directly used in multiple operating systems (including Windows, Linux and Mac OS X), provided that Python and the above listed modules are pre-installed in the system. We also applied PyInstaller (version 2.1) to distribute the MaxReport scripts into standalone executable programs under Windows. MaxReport can be easily used via MaxReport_GUI for those biologists not familiar with computing. And MaxReport_CMD will facilitate the processes of multiple MaxQuant projects using text commands. By defining a specific reporting configuration file, MaxReprot can support different versions of MaxQuant. We have already provided optimized configuration files for several representative versions of MaxQuant (from 1.2.x to 1.5.x) on the homepage of MaxReport website. The parsing rule files for different formats of fasta sequences and correction matrix files for different types of isobaric labelling are also available on this website.

### Application and evaluation of MaxReport

**Fig 2:**
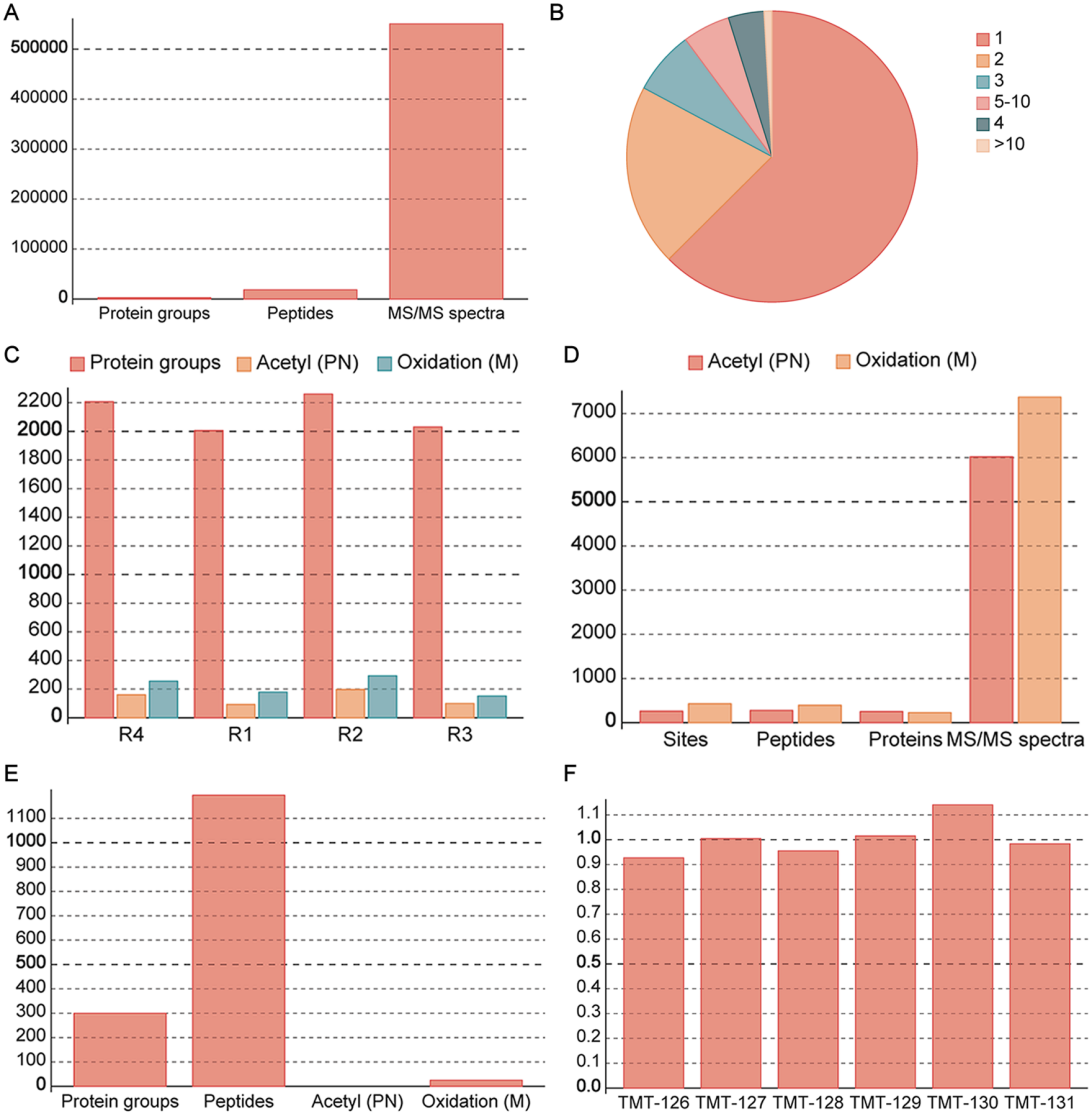
Examples of statistical charts. MaxReport automatically generates various statistical charts for both identification and quantification results including: summary of proteome identification (A); distribution of site counts (B); summary of experiment specific identification (C), PN stands for protein N-terminal. M stands for Methionine residue. R represents different experimental replication; summary of PTM specific identification (D); summary of quantification results (E) and overall calibration factors (F).

We have successfully applied two sample datasets to demonstrate the usage and applicability of MaxReport. One dataset was a qualitative proteome of macaque sperm with four technical replicates [4], while the other dataset was a quantitative Erwinia proteome spiked with four exogenous proteins of known expression ratios [19]. The source results and optimized results of these datasets are also available on the MaxReport website. We showed that MaxReport supports different versions (including 1.2.0.18, 1.2.2.5, 1.3.0.5, 1.4.1.2, 1.5.2.8 and 1.5.3.8) of MaxQuant results for both identification and quantification analyses. The latest version of MaxReport also supports "Ile = Leu" setting and fixes a few bugs of the earlier versions including multiple sequence files selection and creating Excel files with NaN values. The compressed and integrated results were finally exported to a single Excel file for better viewing and sharing. In addition to data compression, MaxReport also provided abundant descriptive statistical analyses and visual figures automatically. As shown in Fig 2, MaxReport generates statistical charts for identification results including: summary of the total number of identified spectra, peptides and proteins (Fig 2A), proportions of proteins with different number of Oxidation sites (Fig 2B), assignment and count of identified proteins and sites in different experimental replications (Fig 2C), and summary of the total number of identified spectra, peptides, proteins and sites for each type of PTM (Fig 2D).

**Fig 3:**
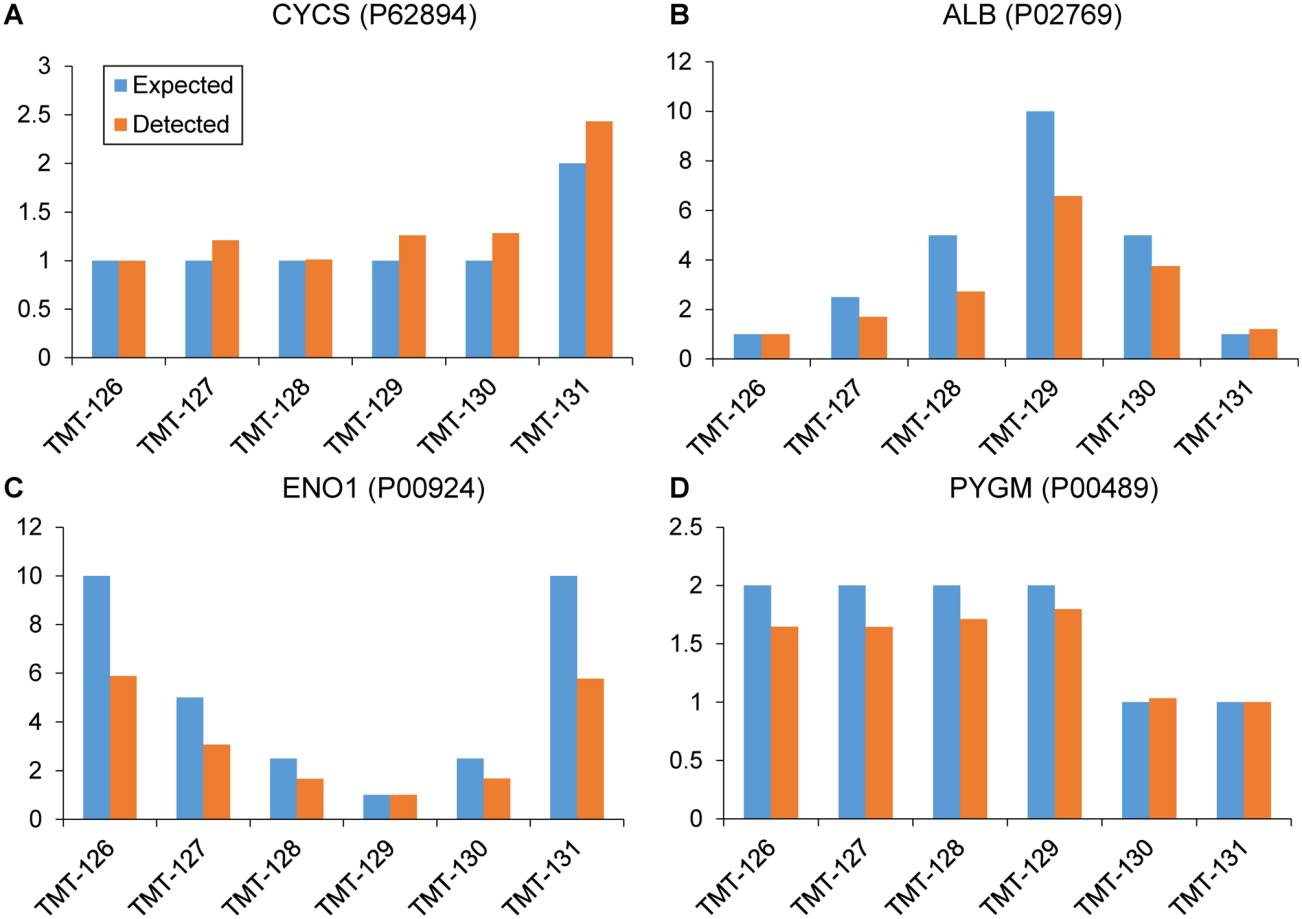
Comparison of the expected ratios with detected ratios. The expected ratios and detected ratios are provided for the four standard proteins: P62894 (A), P02769 (B), P00924 (C) and P00489 (D). The gene name for each protein is also shown in the figure.

For quantification results of the TMT6 dataset, MaxReport calculates the number of quantified proteins, peptides and sites (Fig 2E). It also summarizes the overall calibration factors for each reporter ion to obtain an overview of the experimental bias (Fig 2F). We further compared the expected ratios of the four standards proteins (P62894, P02769, P00924 and P00489) with the detected ratios calculated by Libra algorithm (Fig 3). Considering the intrinsic feature of TMT6 labelling at the MS2 level (the expected ratios were usually underestimated due to complex interference) [23], the detected ratios using Libra algorithm can be used for identification of differentially expressed proteins. Thus, the proposed tool provides enhanced result reporting functions for MaxQuant and is a promising complementary tool to the Perseus (a companion software of MaxQuant) for proteomics users to better understand and share the data.

## Conclusions

Proteomic software plays key role in translating raw data to human-readable results. Many third-party tools can provide enhanced or additional functions for the original proteomic software. In the present study, we developed an enhanced proteomic result reporting tool for MaxQuant, named as MaxReport. MaxQuant is widely used in various types of proteomic studies such as in peptide identification, modification assignment, isotope labelling quantification and label-free quantification. MaxReport can automatically optimize and organize the results of MaxQuant. This tool also provides additional functions including generating visualization figures and exporting integrated results for users who are not familiar with bioinformatics to save time in comprehending and interpreting the results.
